# Wearable Wireless Biosensor Technology for Monitoring Cattle: A Review

**DOI:** 10.3390/ani11102779

**Published:** 2021-09-23

**Authors:** Mingyung Lee, Seongwon Seo

**Affiliations:** Division of Animal and Dairy Sciences, Chungnam National University, Daejeon 34134, Korea; mingyung1203@cnu.kr

**Keywords:** wearable wireless biosensor systems, physiological parameters, sensor performance, meta-analysis, cattle

## Abstract

**Simple Summary:**

The wearable wireless sensor system plays a crucial role in providing behavioral and physiological data for each individual in precision livestock farming. This article reviewed the most types of sensor systems available in the market and summarized detailed information on these systems. Additionally, through meta-analysis, the accuracy of the parameters generated by the sensor system was verified. As a result, it has been shown that there are more than 60 sensor systems of various types have been developed and sold. Most of them generate behavioral and physiological parameters of cattle with excellent performance (e.g., eating time, ruminating time, lying time, standing time, etc.), with the exception of a few parameters (e.g., drinking time and walking time). In this review, it was also investigated that the same parameters predicted by sensor systems of the same brand showed different accuracies, but it was not possible to confirm where this difference originated because the additional experimental conditions presented in the literature were not detailed. Therefore, this review suggested that guidelines for evaluation criteria for research evaluating sensor performance are needed.

**Abstract:**

The review aimed to collect information about the wearable wireless sensor system (WWSS) for cattle and to conduct a systematic literature review on the accuracy of predicting the physiological parameters of these systems. The WWSS was categorized as an ear tag, halter, neck collar, rumen bolus, leg tag, tail-mounted, and vaginal mounted types. Information was collected from a web-based search on Google, then manually curated. We found about 60 WWSSs available in the market; most sensors included an accelerometer. The literature evaluating the WWSS performance was collected through a keyword search in Scopus. Among the 1875 articles identified, 46 documents that met our criteria were selected for further meta-analysis. Meta-analysis was conducted on the performance values (e.g., correlation, sensitivity, and specificity) for physiological parameters (e.g., feeding, activity, and rumen conditions). The WWSS showed high performance in most parameters, although some parameters (e.g., drinking time) need to be improved, and considerable heterogeneity of performance levels was observed under various conditions (average *I*^2^ = 76%). Nevertheless, some of the literature provided insufficient information on evaluation criteria, including experimental conditions and gold standards, to confirm the reliability of the reported performance. Therefore, guidelines for the evaluation criteria for studies evaluating WWSS performance should be drawn up.

## 1. Introduction

To increase the sustainability of the dairy industry, there has been an increased need for replacing traditional group-level management with precision dairy farming, which continuously monitors and manages individual productivity and health issues [1]. However, individual monitoring through direct observation of farm staff or video recordings is time-consuming, labor-intensive, difficult to detect accurately, and practically impossible on large-sized farms. Therefore, wearable wireless biosensor systems have been introduced for individual cow monitoring, and research on these systems has been actively conducted in the last 40 years [2].

The wearable wireless biosensor system is composed of a battery, a data transmitter, and one or more sensors (tri-axis accelerometer, thermometer, pH electrode, microphone, etc.), which are mounted on the cow’s body to measure and collect biometric data. These sensors can be divided into eight types (ear tags, halters, neck collars, reticulo-rumen bolus sensors, leg tags, tail tags, tail head tags, and vaginal tags) according to their location on the dairy cow’s body [3]. They are used to collect and transmit biometric data, such as acceleration, temperature, pH, and pressure at specified time intervals. These raw data from the sensors are then computed into physiological and behavioral parameters (such as the number of steps, activity level, time spent for eating, ruminating, or lying) by algorithms in the sensor, by the PC software, or through clouding computing. Additionally, these parameters are used as the predictor variables for the diagnosis model for detecting physiological and health status (e.g., estrus events, calving, and illness).

The literature reviews about cattle biosensor systems have primarily focused on the performance of diagnostic models and detection alarms [2,4]. However, the parameters generated from the sensors are important, not only for ensuring high performance of the detection alarms of their diagnostic models, but also for obtaining ‘big data’ of physiological status and behavior of individual cattle. Therefore, it is important to investigate how accurately the parameters generated from the sensors can represent animal physiological and behavioral parameters. Thus, the purposes of this review paper were to (1) collate commercially available wearable wireless biosensor systems for cattle farms and (2) review the literature focused on evaluating the accuracy of the parameters obtained from these biosensor systems in predicting the actual condition of animals.

## 2. Currently Available Wearable Wireless Biosensor Systems

### 2.1. Search Strategy and Quality Evaluation of the Constructed Database

In this review, we collected all the information about the currently available wearable wireless biosensors for cattle, summarizing the basic features of these sensors. Our comprehensive search was performed through a web-based search on Google, and the search terms were as follows:

cattle AND sensor AND (ear OR halter OR neck OR rumen OR leg OR tail OR vagina)

The inclusion criterion was that the product must be currently commercially available. The availability of the sensors was confirmed based on the information obtained from the respective web pages. The products marked as ‘in development’ or ‘to be released soon (concept solutions and prototypes)’ were excluded from this study, i.e., only the products currently available in the market were included in the study. The initial search lasted for three months (August 2019~October 2019). It was conducted extensively and meticulously to obtain a comprehensive market inventory and minimize the risk of missing any relevant products. While writing this review, the search process was re-conducted to prevent the omission of newly released products (~April 2020). During this iterative process, we double-checked if there were any missing products in the existing database.

Technical specifications and information on vendor websites were our primary sources of information, and business reports and research papers were additional sources for this review. If we found any further information about a product in scientific articles, we used this information to update our product information. For an objective evaluation of database quality, our database was compared with another independent database, the sensor product database for dairy cattle provided by the Data Driven Dairy Decision for Farmers (4D4F) project (https://www.4d4f.eu/, last updated on 23 August 2019) funded from the European Union’s Horizon 2020 research and innovation program.

### 2.2. Wearable Wireless Biosensor Systems by Type and Mounting Location

#### 2.2.1. Ear Tag and Halter Type

Several wearable wireless biosensors that can be mounted externally on the animal body, such as on ears, necks, legs, and tails, have been developed. Among these, ear-mounted sensors are mainly equipped with sensors that measure temperature and activity. They are mostly mounted in the middle of the ear and used to check the animals’ health status using temperature data. Most ear tag products equipped with three-axis accelerometer sensors can additionally check the animal’s ruminating, eating, resting, and activity. The management system connected to the sensor uses these data to diagnose an animal’s estrous cycle and health issues.

Halter type sensors are attached to the cow’s head, and they measure the cow’s eating and ruminating behavior through a noseband pressure sensor and a three-axis accelerometer sensor. The currently available ear tag and halter type sensors are listed in Table 1 and Table 2.

#### 2.2.2. Neck Collars

The neck collar sensor system consists of a device with sensors attached to the strap hanging on a cow’s neck. This type of sensor is the most commonly used in dairy farms; many companies manufacture it. Generally, neck collars have been widely used to control the amount of feed or measure individual feed intake through radio-frequency identification technology. Recently, accelerometer and microphone sensors have been added to neck collars to measure eating time, rumination time, and activity level. Some are equipped with temperature sensors to measure an animal’s body temperature. These sensors provide farm managers with a cow’s health and estrus information. Some neck collar sensors are used in combination with automatic milking systems. The currently available commercial neck collar tag sensors are listed in Table 3 and Table 4.

#### 2.2.3. Reticulo-Rumen Bolus Sensors

A rumen bolus system is inserted orally and placed in the reticulum, where it will remain throughout the animal’s life. It is designed to continuously monitor a few rumen parameters (temperature and pH) and an animal’s activity throughout the day. The bolus is equipped with an internal battery, a temperature sensor/pH sensor/accelerometer, and a transmitter for data transmission. Its battery can last for months to years and can transmit the data wirelessly at adjustable time intervals.

Bolus sensors are primarily designed to sense ruminal temperature changes, which can signal a shift in animal physiological states. A decrease in ruminal temperature reflects drinking and eating events, and its increase coincides with increased body temperature [5,6,7]. Monitoring changes in the ruminal temperature and activity can facilitate early detection of abnormal behavior, estrous cycle, and illnesses. Unfortunately, the pH sensor is mostly unequipped due to its relatively short lifespan. The currently available commercial bolus sensor systems with a pH sensor have an operational lifetime of no more than a few months since the stability of the pH probe is limited. Thus, rumen bolus systems with a pH sensor are mainly considered as research tools. The currently available commercial bolus products are presented in Table 5 and Table 6.

#### 2.2.4. Leg Tags

Along with neck collar sensors, leg tag sensors are a popular sensor technology used in farms. Leg tag sensors are mainly equipped with three-axis accelerometers, which can measure animal activity, walking time, lying time, standing time, and the number of steps. They also provide farm managers with a cow’s health and estrus information. Similar to the neck collar system, some leg tag sensors are used in combination with automatic milking systems. The currently available commercial leg tag products are presented in Table 7 and Table 8.

**Table 1:** Information about currently available ear-tag and halter type sensor.

Product	Company (Parent Company)	Country	Management Software	Mobile Application	Dimensions (mm × mm × mm)	Weight (g)	Battery Life	Range (m)	Built-in Sensors
Ear tag									
Smartbow	Smartbow GmbH (Zoetis Services LLC.)	AT	Herd Monitoring Software	○	52 × 36 × 17	34	2 years	300	Accelerometer Temperature sensor
eSense Flex tag	SCR Engineers Ltd. (Allflex Europe SA)	IL	SenseHub™/ Heatime^®^ Pro+	○	68 × 38 × 15	25	3 years	200 × 500 *	Accelerometer
CowManager SensOor	Agis Automatisering BV	NL	CowManager System	○	60 × 50 × 22	32	5 years	-	Accelerometer Temperature sensor
TekSensor tag	TekVet Technologies Co.	NL	TekAccess™	×	-	-	-	-	Temperature sensor
Calf Tag	FeverTags LLC	US	TempVerified	×	-	14	2 years	-	Temperature sensor
Data Collection Tag	FeverTags LLC	US	-	×	-	-	-	-	Temperature sensor
Halter									
RumiWatch Noseband Sensor	ITIN + HOCH GmbH	CH	RumiWatch Manager/RumiWatch Converter	×	-	-	2 years	-	Accelerometer Temperature sensorPressure sensor

* Area coverage.

**Table 2:** Output data and detection items of the wearable wireless biosensor systems (ear tag and halter type).

Product (Module)	Output Data	Data Reporting Frequency	Detection
Ear tag			
Smartbow	High activity/Activity/Inactivity/Ruminating time/Location	Every hour	Heat/Health disorder
eSense™ Flex tag	Activity/Ruminating time/Heat index	Every 2 h	Heat/Health disorder
CowManager SensOor (Find my cow)	High activity/Activity/Inactivity/Ruminating time/Eating time/Temperature/(Location)	Every hour	Heat/Health disorder
TekSensor tag	Temperature	Every hour	Health disorder
Calf Tag	Temperature	Every 15 min	Health disorder
Data Collection Tag	Temperature	Every 15 min	Research purpose (Data acquisition only)
Halter			
RumiWatch Noseband Sensor	Raw activity/Other chewing activity/Ruminating time/Regurgitated boli counts/Ruminating chew counts/Chews per bolus/Chews per minute/Eating time/Eating chew counts/Drinking time/Drinking gulp count/Temperature (ambient)	Every minute /Every hour	Research purpose (Data acquisition only)

**Table 3:** Information about currently available neck collar type sensor.

Product	Company (Parent Company)	Country	Management Software	Mobile Application	Dimensions (mm × mm × mm)	Weight (g)	Battery Life	Range (m)	Built-In Sensors
CowScout Neck	GEA Farm Technologies, Inc.	DE	CowScout Activity monitoring system	○	-	-	5 years	100–1000	Accelerometer
Rescounter III Neck	GEA Farm Technologies, Inc.	DE	DairyPlan C21	×	-	-	-	-	Accelerometer
Axel Collar	Medria Inc.	FR	Farm’Life^®^	○	100 × 48 × 30	160	-	1000	Accelerometer
Smart Collar	HerdInsights	IE	HerdInsights Software	○	-	-	5 years	-	Accelerometer
Moocall Heat	Moocall Ltd.	IE	Moocall Breedmanager	○	-	-	60 days	3G coverage	-
MooMonitor+	Dairy Master	IE	Dairymaster MooMonitor	○	-	-	10 years	1000	Accelerometer
SmartTag Neck	Pearson International LLC	IE	Pearson Heat Detection with Health Monitoring system	○	-	-	10 years	-	Accelerometer
cSense Flex tag	SCR Engineers Ltd. (Allflex Europe SA)	IL	SenseHub™ Dairy/SenseHub™ Beef/ Heatime^®^ Pro+ System	○	84 × 64	98	7 years	200 × 500 *	Accelerometer
SCR H-LD	SCR Engineers Ltd. (Allflex Europe SA)	IL	Heatime^®^ HR System (Independent device)/ Heatime^®^ Pro+ System (PC)	○	84.1 × 64.5	98	7 years	200 × 500 *	Accelerometer
SCR HR-LD/SCR HR-LDn	SCR Engineers Ltd.(Allflex Europe SA)	IL	Heatime^®^ HR System(Independent device)/Heatime^®^ Pro+ System (PC)	○	84.1 × 64.5	98	7 years	200 × 500 *	Accelerometer/Microphone
Qwes ISO LD/LD Smarttag	Lely	IL	Lely T4C management system	○	-	-	-	75	Accelerometer
Qwes H-LD	Lely	IL	Lely T4C management system	○	-	-	-	500	Accelerometer
Qwes HR-LDn	Lely	IL	Lely T4C management system	○	-	-	-	500	Accelerometer/Microphone
AfiCollar	Afimilk Agricultural Cooperative Ltd.	IL	AfiFarm Software/Afi2Go Pro Mobile App	○	-	-	-	200–800	Accelerometer
Milkrite|InterPuls Neck Tag	milkrite | InterPuls	IT	MyFarm	○	-	-	-	75–500	Accelerometer
Smarttag Neck	Nedap livestock management	NL	Nedap CowControl	○	-	-	10 years	75	Accelerometer
Smarttag Neck/All in One	CRV international B.V.	NL	Ovalert	○	-	-	-	-	Accelerometer
Activity meter system	DeLaval International AB Inc.	SE	AlPro/DelPro Farm Management systems	○	-	170	10 years	200	Accelerometer
Cowlar	Cowlar	US	Cowlar	×	110 × 62 × 33	242	6 months	>3000	Accelerometer/Temperature sensor
HeatSeeker II Neck	BouMatic LLC	US	HerdMetrix™	○	-	135	7 years	100–750	Accelerometer
RealTime SmartTag	BouMatic LLC	US	HerdMetrix™	○	-	-	-	-	Accelerometer

* Area coverage.

**Table 4:** Output data and detection items of the wearable wireless biosensor systems (neck collar type).

Product (Module)	Output Data	Data Reporting Frequency	Detection
CowScout Neck	Activity/Inactivity/Ruminating time/Eating time	Every 2 h	Heat/Health disorder
Rescounter III Neck	Activity	Every 2 h	Heat
Axel Collar (Feed’Live/Heat’Live/Time’Live)	High activity/Inactivity/Ruminating time/Eating time/Lying time/Standing time	-	Heat/Health disorder
Smart Collar	Activity/Inactivity/Ruminating time/Eating time/Heat index	Every hour	Heat/Health disorder
Moocall Heat	-	-	Heat
MooMonitor+	High activity/Activity/Low activity/Inactivity/Ruminating time/Eating time	Every hour	Heat/Health disorder
SmartTag Neck	Eating time/Not eating time	-	Heat/Health disorder
cSense Flex tag	Activity/Ruminating time/Heat index	Every 2 h	Heat/Health disorder
SCR H-LD	Activity/Heat index	Every 2 h	Heat/Health disorder
SCR HR-LD /SCR HR-LDn	Activity/Ruminating time/Heat index	Every 2 h	Heat/Health disorder
Qwes ISO LD	Activity	Every 2 h	Heat
Qwes ISO LD Smarttag (CowLocator)	Activity/Ruminating time/(Location)	Every 2 h	Heat/Health disorder
Qwes H-LD	Activity	Every 2 h	Heat
Qwes HR-LDn	Activity/Ruminating time	Every 2 h	Heat/Health disorder
AfiCollar	Activity/Ruminating time/Eating time	-	Heat/Health disorder
Milkrite|InterPuls Neck Tag	Activity/Ruminating time/Eating time/Location	-	Heat/Health disorder
Smarttag Neck (Cow positioning)	Activity/Inactivity/Ruminating time/Eating time/Eating bouts/(Location)	Continuously	Heat/Health disorder
Smarttag Neck	Eating time/Not eating time	Continuously	Heat/Health disorder
Smarttag All in One (Cow positioning)	Inactivity/Ruminating time/Eating time/Not eating time/(Location)	Continuously	Heat/Health disorder
Activity meter system	Activity/Heat index	Every hour	Heat/Health disorder
Cowlar	Activity/Ruminating time/Eating time/Step counts	-	Heat/Health disorder
HeatSeeker II Neck	Activity	Every 2 h	Heat
RealTime SmartTag (Activity/Rumination & Localization)	Activity/Inactivity/Ruminating time/Eating time/(Location)	Every 2 h	Heat/Health disorder

**Table 5:** Information about currently available rumen bolus type sensors.

Product	Company (Parent Company)	Country	Management Software	Mobile Application	Dimensions (mm × mm × mm)	Weight (g)	Battery Life	Range (m)	Built-In Sensors
smaXtec classic/ pH Plus Bolus	smaXtec Animal Care Inc.	AT	smaXtec Messenger 4.0	○	105 × 35 132 × 35	-	4 years	10–30	Accelerometer/Temperature sensor/ (pH sensor)
San’Phone	Medria Inc.	FR	Farm’Life^®^	○	-	-	-	1000	Temperature sensor
Moow Rumen Bolus	Moow Farm Ltd.	HU	Moow system	○	-	-	3 years	-	Temperature sensor/ pH sensor
Smart Rumen Bolus (Temp/ Temp + Activity/ Temp + Activity +pH)	Moonsyst Industrial Technologies Ltd.	HU	Moonsyst system	○	-	-	6 years	-	Temperature sensor/(Accelerometer)/ (pH sensor)
LiveCare	uLikeKorea Co., Inc.	KR	Livestock HealthCare Services	○	110 × 25	-	6 years	-	Accelerometer/Temperature sensor/ (pH sensor)
eBolus	eCow Ltd.	UK	eCow Software	×	135 × 27	150	5 months	Handheld antenna	Temperature sensor/ pH sensor
HerdStrong	DVM Systems Co.	US	HerdStrong^®^ Tru-Core system	○	114 × 33 × 31	-	5 years	137	Temperature sensor
SmartStock	Smart Stock Ltd.	US	Healthy Cow Dairy	×	85 × 30	120	5 years	91–182	Temperature sensor

**Table 6:** Output data and detection items of the wearable wireless biosensor systems (rumen bolus type).

Product (Module)	Output Data	Data Reporting Frequency	Detection
smaXtec classic/pH Plus Bolus	Activity/Temperature/(pH)	Every 10 min	Heat/Health disorder/Calving
San’Phone	Temperature	-	Research purpose (Data acquisition only)
Moow Rumen Bolus	Temperature/pH	-	Health disorder
Smart Rumen Bolus (Temp/Temp + Activity/Temp + Activity +pH)	Activity/Temperature/(pH)	-	Heat/Health disorder
LiveCare	Activity/Drinking bouts/Temperature/(pH)	Every hour	Heat/Health disorder/Calving
eBolus	Temperature/(pH)	Every 15 min	Research purpose (Data acquisition only)
HerdStrong	Temperature	Every 15 min	Heat/Health disorder/Calving
SmartStock	Temperature	Customizable	Health disorder

**Table 7:** Information about currently available leg-tag type sensor.

Product	Company (Parent Company)	Country	Management Software	Mobile Application	Dimensions (mm × mm × mm)	Weight (g)	Battery Life	Range (m)	Built-In Sensors
Rumiwatch pedometer	ITIN + HOCH GmbH	CH	RumiWatch Manager/RumiWatch Converter	○		-	2 years	-	Accelerometer/Temperature sensor
CowScout Leg	GEA Farm Technologies, Inc.	DE	CowScout Activity monitoring system	○	-	-	5 years	100–1000	Accelerometer
Rescounter III Leg	GEA Farm Technologies, Inc.	DE	DairyPlan C21	×	-	-	-	-	Accelerometer
IceTag/IceQube (for research)	IceRobotics Ltd.	UK	IceReader & IceManager	×	65 × 60 × 30 96 × 81 × 31	130	2 years	-	Accelerometer
IceQube	IceRobotics Ltd.	UK	CowAlert	○	96 × 81 × 31	130	2 years	-	Accelerometer
Breeder Tag	Genus Breeding Ltd.	UK	Breeder Tag System	○	-	-	5 years	700	Accelerometer
SmartTag Leg	Pearson International LLC	IE	Pearson Heat Detection with Health Monitoring system	○	-	-	10 years	-	Accelerometer
AfiAct II	Afimilk Agricultural Cooperative Ltd.	IL	AfiFarm Software/Afi2Go Pro Mobile App	○		-	5 years	200–800	Accelerometer
Track A Cow	ENGS Systems	IL	EcoHerd Software	×	68 × 50 × 26	124	6 years	700–2000	Accelerometer
milkrite|InterPuls Pedometer	milkrite | InterPuls	IT	MyFarm	○	-	-	-	75–1000	Accelerometer
Gyuho (cow step) SaaS	Fujitsu	JP	Gyuho SaaS system	×	-	-	-	-	Accelerometer
Smarttag Leg	Nedap livestock management	NL	Nedap CowControl	○	-	-	10 years	75	Accelerometer
Smarttag Leg	CRV international B.V.	NL	Ovalert	○	-	-	-	-	Accelerometer
HeatSeeker II Leg	BouMatic LLC	US	HerdMetrix™	○	-	135	7 years	50	Accelerometer

**Table 8:** Output data and detection items of the wearable wireless biosensor systems (leg-tag type).

Product (Module)	Output Data	Data Reporting Frequency	Detection
Rumiwatch pedometer	Raw activity/Lying time/Standing time/Walking time/Stand up bouts/Lie down bouts/Step counts/Temperature (ambient)	Every minute/Every hour	Research purpose (Data acquisition only)
CowScout Leg	Activity/Lying time/Standing time/Walking time/Stand up bouts/Step counts	Every 2 h	Heat/Health disorder
Rescounter III Leg	Activity	Every 2 h	Heat
IceTag/IceQube (for research)	Activity/Lying time/Standing time/Stand up bouts/Lie down bouts/Step counts	Customizable	Research purpose (Data acquisition only)
IceQube	Activity/Lying time/Standing time/Stand up bouts/Lie down bouts/Step counts	Every 15 min	Heat/Health disorder
Breeder Tag	Activity/Lying time/Step counts	Every 15 min	Heat/Health disorder
SmartTag Leg	Inactivity/Lying time/Standing time/Step counts	-	Heat/Health disorder
AfiAct II	Lying time/Lie down bouts/Step counts	Every hour	Heat/Health disorder/Calving
Track A Cow	Lying time/Standing time/Step counts	Every 6 min	Heat/Health disorder
milkrite|InterPuls Pedometer	Activity/Lying time/Standing time/Walking time/Stand up bouts/Step counts	NA	Heat/Health disorder
Gyuho (cow step) SaaS	Step counts	Every hour	Heat
Smarttag Leg	Activity/Lying time/Standing time/Walking time/Stand up bouts/Step counts	Continuously	Heat/Health disorder
Smarttag Leg	Lying time/Stand up bouts/Step counts	Continuously	Heat/Health disorder
HeatSeeker II Leg	Activity	Every 2 h	Heat

#### 2.2.5. Tail and Vagina Mounted Types

Both dystocia and stillbirth significantly impact on animal productivity and farm profitability, often requiring a skilled assistant and immediate intervention at the moment of delivery [8]. In order to reduce the reliance on labor and aid animal management, sensors detecting the calving time without physical observation have been developed. These sensors are attached to the tail (or tail head), and they measure tail movement patterns triggered by labor contractions.

Among the sensors used to detect calving, some sensors are inserted directly into a cow’s vagina. Using the principle that a cow’s body temperature decreases before calving [9,10,11], vaginally inserted sensors detect a reduction in a cow’s vaginal temperature and provide a calving alarm to farm managers. Another type of vaginally inserted sensor detects light. When the device is pushed out of the vagina by a cow’s water break, it is recognized that the device is out of the cow’s body through detecting light. At this time, the device sends a text message to the farm manager to notify the start of calving. The currently available commercial products of the abovementioned types are presented in Table 9.

## 3. Literature Review on the Evaluation of Parameters Generated by Wearable Wireless Biosensor Systems

Wearable wireless wearable biosensors provide farm managers with physiological and behavioral data, such as eating, rumination, walking, and lying time. These data are generated by computing raw data measured by the sensor using a specific algorithm. The units of the generated values depend on the sensor type and the algorithm used. As the computed physiological and behavioral parameters are used as predictor variables in health and estrus diagnostic models, they should accurately represent the actual state of individual animals. Several studies have been conducted to verify the performance of different sensors. The majority of these studies conducted correlation analyses between the sensor data and the gold standard (actual observations) and performance analyses (i.e., sensitivity, specificity, accuracy, and precision). We reviewed the literature on the evaluation of physiological and behavioral data generated by wearable wireless biosensors.

### 3.1. Search Strategy, Study Selection, and Quality Assessment

A literature search was conducted by a keyword search in Scopus. To avoid an excessive number of search results, we used specific keywords. The final query used to search for articles in the databases was (TITLE-ABS-KEY (correlation OR correlated OR regression OR sensitivity OR specificity OR precision OR accuracy)) AND (TITLE-ABS-KEY (cow OR cattle OR calf OR heifer OR buffalo)) AND ((TITLE-ABS-KEY (sensor* AND NOT sensory)) OR (TITLE-ABS-KEY (automat* OR *meter OR device OR tag))) AND (TITLE-ABS-KEY (detect* OR monitor* OR record*)) AND NOT (TITLE-ABS-KEY (genetic* OR chromatography OR follicle OR muscle OR meat OR DNA OR antibody OR serum OR patient OR assay OR spectro*)) AND (LIMIT-TO (DOCTYPE, ‘ar’)) AND (LIMIT-TO (LANGUAGE, ‘English’)). A total of 1875 articles were retrieved using this query (search date: 26 April 2020).

After the initial database search was completed, we screened the title and abstract of each selected article and made decisions on the suitability of each study for inclusion in this review. Articles were included in the final database if they (i) investigated the performance of wearable wireless biosensors for beef or dairy cattle, (ii) evaluated variables related to feeding behavior, moving behavior, or rumen status generated by the sensors, (iii) tested the performance of the sensors with other independent reference measurements (a.k.a. the gold standard), such as real-time or recorded visual observations for the behavioral activities and manual pH or temperature measurements, and (iv) presented at least one or more quantitative evaluation measures, such as correlation, accuracy, precision, sensitivity, and specificity. A total of 46 articles met the above criteria and were selected for our systematic review. These studies evaluated the sensor’s performance in monitoring the following three parameters: feeding behavior, activity behavior, and rumen status. The following information was extracted from the selected papers: target behavioral and physiological parameter (i.e., feeding behavior: eating time, ruminating time, drinking time; activity behavior: lying time, standing time, walking time, step count, active time, inactive time; rumen statue: rumen pH and rumen temperature), sensor information (i.e., mounting position, product name, company, country), animal information (i.e., breed, gender, physiological stage), housing information (i.e., barn type, feeding method), gold standard information (i.e., method, number of observers, reliability between observers), data quantity (i.e., number of animals, total collection time, mean collection time per animal), and evaluation results (i.e., correlation coefficient: Pearson, Spearman, Concordance; diagnostic accuracy: sensitivity, specificity, precision, accuracy).

### 3.2. Evaluation of Wearable Wireless Biosensor Systems

In this study, feeding behavior was classified as eating, ruminating, or drinking. Feeding behavior is usually measured by a sensor located on the head of the cow, such as an ear tag, halter, or neck collar. Activity behavior was classified as lying, standing, walking, active, or inactive (resting). These activities are usually measured by leg tag sensors; however, there are other types of sensors (e.g., ear tags and neck collars) capable of recording daily active and inactive time. As the gold standard for evaluating the sensor, the total duration of the target behavior quantified through visual observation of an observer is used for the behavioral activities, while independent measurements are used for physiological parameters (rumen pH and temperature). During observation, the trained observer records the start time and end time of the target behavior and calculates the duration of target behavior based on this record. The target behavior is defined through an ethogram, and the observer is trained to identify the animal’s behavior based on this definition before observation. Visual observation of an observer includes both real-time (live observation) and non-real-time (video recordings) observations. The case where values derived from other wearable wireless sensors were used as the gold standard were excluded from this study.

The correlation results, i.e., the values of Pearson’s correlation coefficient (PCC), Spearman’s rank correlation coefficient (SCC), and Lin’s concordance correlation coefficient (CCC) were graded using the criteria of Hinkle et al. [12]. The grades were negligible (0.00–0.30), low (0.30–0.50), moderate (0.50–0.70), high (0.70–0.90), and very high (0.90–1.00). PCC and SCC can describe a linear relationship between a measured value and a value to be compared, and CCC can additionally explain the degree of agreement with the measured value as well as the linear relationship. In this review, along with correlation and CCC, the results of binary classification tests based on 2 × 2 contingency tables (true positives, false negatives, false positives, and true negatives) of the sensors presented in the articles are also discussed. The following performance results were considered: sensitivity (*Se*; true positives out of the sum of true positives and false negatives), specificity (*Sp*; true negatives out of the sum of true negatives and false positives), accuracy (*Acc*; true positives and true negatives out of the total number of tests), and precision (*Pre*; true positives out of the sum of true positives and false positives; positive predictive value).

### 3.3. Statistical Analysis

A meta-analysis was performed for the reported correlation coefficients (PCC, SCC, and CCC) and diagnostic accuracy (i.e., *Se* and *Sp*). The mean and 95% confidence intervals of the statistics were estimated through a random-effects model based on the DerSimonian–Laird estimator [13], which was generally considered as the standard procedure in the meta-analysis. Since the animal types, physiological stages of animals, feeding and housing conditions, and sensor products were varied among the studies included in the meta-analysis, the random-effects model was selected instead of a fixed-effects model. Given the non-normality of correlation coefficients, point estimates were variance-stabilized using Fisher’s z-transform [14]. The mean value from each study was weighted based on the inverse variance method using the study sample size (number of animals). We treated evaluations conducted under different conditions within the same article as separate individual studies. The analysis was not performed if there were no more than two independent study samples for one behavior. Heterogeneity was examined using *τ*^2^, *I*^2^, and Cochran’s Q statistic, where *τ*^2^ = 0 suggests no heterogeneity, and *I*^2^ values of 25, 50, and 75% correspond to cut-off points for low, moderate, and high heterogeneity, respectively [15]. The differences in the correlation between sensor types were analyzed using analysis of variance. All the procedures of the meta-analysis were performed using the ‘metacor’ function in the ‘meta’ package of R version 4.0.3 [16]. Statistical significance was set at *p* < 0.05, and the results characterized by 0.05 ≤ *p* < 0.1 were considered trends.

#### 3.3.1. Feeding Behavior

##### Eating Time

**Figure 1:**
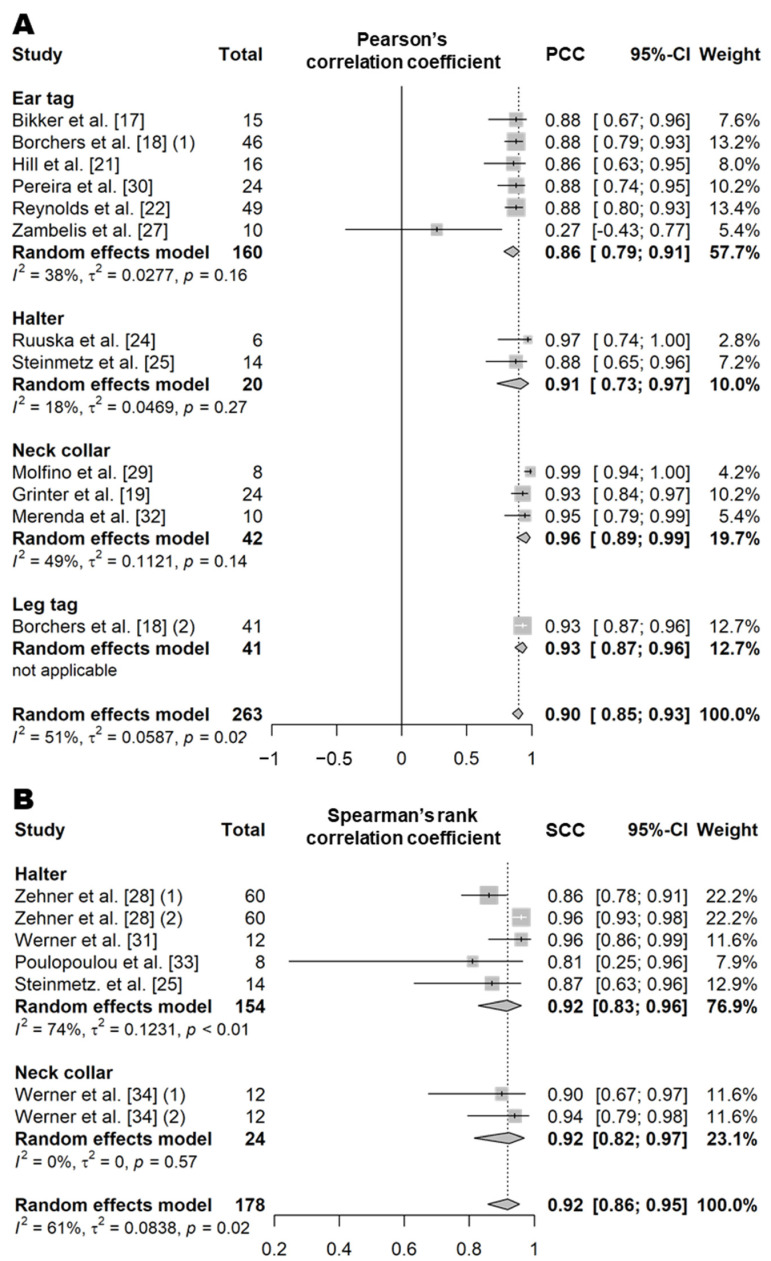
Forest plots of the correlation coefficient of eating time between wearable sensors and visual observation. (**A**,**B**) show Pearson’s correlation coefficient and Spearman’s correlation coefficient, respectively. Numbers in parentheses indicate individual studies applying different evaluation conditions within the same article. ‘Total’ means the sample size of each study and ‘Weight’ means the weight for the mean based on the sample size.

**Figure 2:**
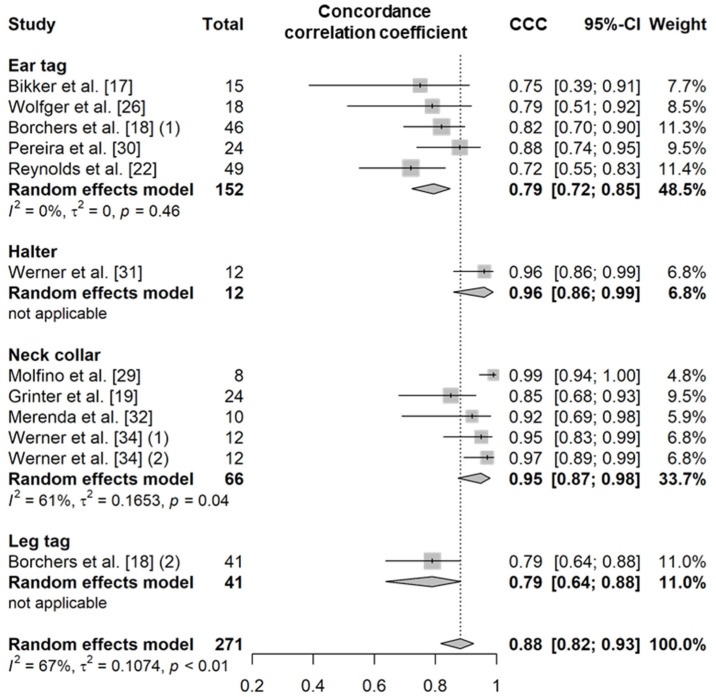
Forest plot of the concordance correlation coefficient of eating time between wearable sensors and visual observation. Numbers in parentheses indicate individual studies applying different evaluation conditions within the same article. ‘Total’ means the sample size of each study and ‘Weight’ means the weight for the mean based on the sample size.

Eating time refers to the amount of time that an animal spends consuming feed per day. This variable was evaluated in both indoor intensive farming systems (such as free-stall barns or tie-stall barns) and pasture systems (Supplementary Tables S1 and S2). In intensive farming, eating behavior is defined as the chewing or licking movement occurring when the animal’s muzzle is located in or above the feed bunk [17,18,19,20,21,22,23,24,25,26,27,28]. In pasture systems, eating behavior is defined as the process of biting or chewing grass when the cow’s muzzle is located near or above the grass [29,30,31,32,33,34]. PCC and SCC values based on 18 independent study samples from 15 articles (12 for PCC and seven for SCC) showed that the correlation between the eating time recorded by sensors and actual observations was very high, regardless of the sensor type (PCC = 0.90, *n* = 263, *I*^2^ = 51%; SCC = 0.92, *n* = 178, *I*^2^ = 61%; Figure 1 and Supplementary Table S1) [17,18,19,21,22,24,25,27,28,29,30,31,32,33,34]. Moreover, the CCC value based on 12 independent study samples from 10 articles was high (0.88, *n* = 271, *I*^2^ = 67%; Figure 2 and Supplementary Table S1) [17,18,19,22,26,29,30,31,32,34]. The sensor products used between the studies were the same except for the neck collar type (Supplementary Table S1), and the animal type and feeding method were different but showed moderate heterogeneity overall (*I*^2^ = 60% and τ^2^ = 0.25). Among the different types of sensors, on average, the eating time measured by the halters and neck collar tags showed higher correlation with the visual observations (halters, PCC = 0.91 and CCC = 0.96 [24,25,26,31,33]; and the neck collars, PCC = 0.96 and CCC = 0.95 [18,28,31,33]) than that measured by the ear tag sensors (PCC = 0.86, *p* = 0.07; and CCC = 0.79, *p* < 0.01) [17,18,21,22,26,27,30]. The results of a binary classification test for the performance of sensors for eating time obtained from 10 independent study samples from seven articles (10 for *Se*, nine for *Sp*, seven for *Acc*, and nine for *Pre*; Table 10 and Supplementary Table S2) showed an *Se* of 85% (*n* = 220), an *Sp* of 96% (*n* = 210), an *Acc* of 91% (*n* = 184), and a *Pre* of 87% (*n* = 210) [20,23,25,26,28,29,32].

##### Rumination Time

**Figure 3:**
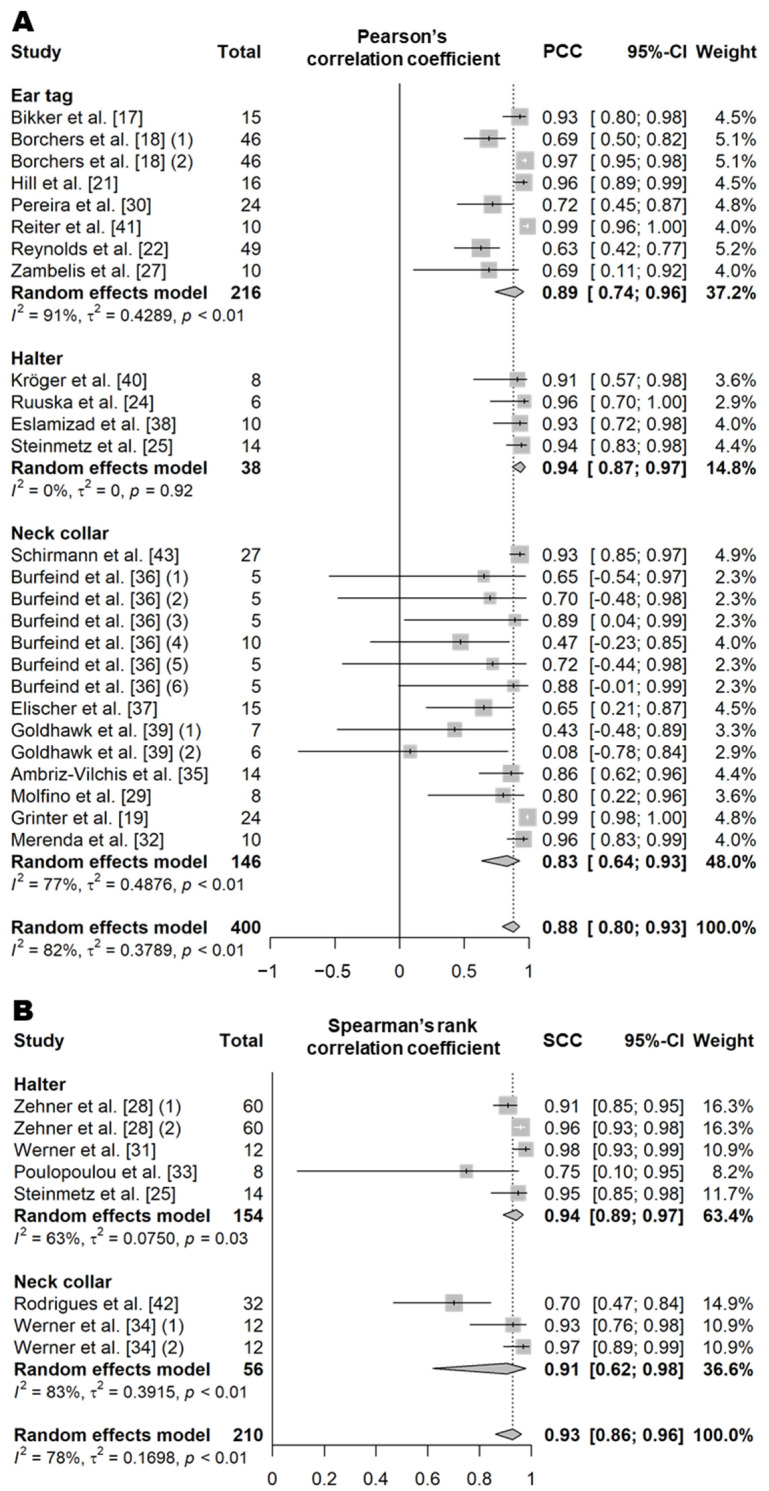
Forest plot of the correlation coefficient of rumination time between wearable sensors and visual observation. (**A**,**B**) show Pearson’s correlation coefficient and Spearman’s correlation coefficient, respectively. Numbers in parentheses indicate individual studies applying different evaluation conditions within the same article. ‘Total’ means the sample size of each study and ‘Weight’ means the weight for the mean based on the sample size.

**Figure 4:**
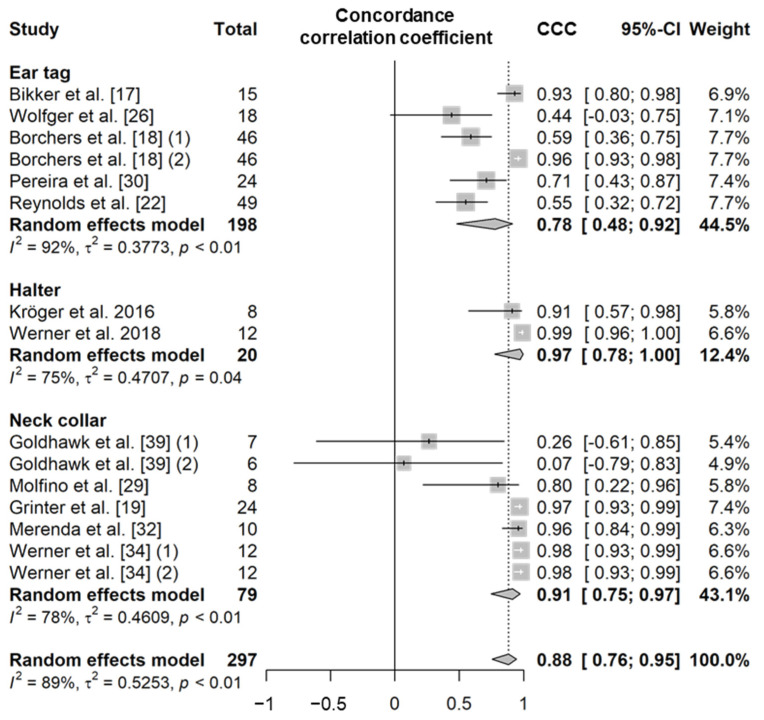
Forest plot of the concordance correlation coefficient of rumination time between wearable sensors and visual observation. Numbers in parentheses indicate individual studies applying different evaluation conditions within the same article. ‘Total’ means the sample size of each study and ‘Weight’ means the weight for the mean based on the sample size.

Rumination time is a variable that represents the amount of time a cow spends ruminating per day. In the literature, ruminating behavior is defined as a behavior that includes regurgitation, rhythmic chewing, and swallowing of the bolus [17,18,19,20,21,22,23,24,25,26,27,28,29,30,31,32,33,34,35,36,37,38,39,40,41,42,43]. PCC and SCC values based on 33 independent study samples from 25 articles (26 for PCC and eight for SCC; Supplementary Table S3) showed that the rumination time recorded by sensors was highly correlated with visual observations regardless of the sensor type (PCC = 0.88, *n* = 400, *I*^2^ = 82%; SCC = 0.93, *n* = 210, *I*^2^ = 78%; Figure 3) [17,18,19,21,22,24,25,27,28,29,30,31,32,33,34,35,36,37,38,39,40,41,42,43]. The CCC value based on 15 independent study samples from 12 articles was also high (0.88, *n* = 297, *I*^2^ = 89%; Figure 4) [17,18,19,22,26,29,30,31,32,34,39,40]. The sensor products, animal types, and feeding methods used were all varied between studies included in the meta-analysis (Supplementary Table S3), and as a result, overall high heterogeneity was observed (*I*^2^ = 83% and τ^2^ = 0.36). The data recorded by the halter sensors showed a very high correlation with the actual observed durations of rumination time (PCC = 0.94, SCC = 0.94, and CCC = 0.97) [20,24,25,28,31,33,38,40]; similarly, the data from the ear tag and neck collar sensors showed a high correlation with the actual observed durations of rumination time (ear tag, PCC = 0.89 and CCC = 0.78 [17,18,21,22,26,27,30,41]; and neck collar, PCC = 0.83, SCC = 0.91, and CCC = 0.91 [19,29,32,34,35,36,37,39,42,43]) (Supplementary Table S3). However, there was no significant difference in the correlation between the sensor data and the visual observation data of rumination time among the different sensor types (*p* > 0.05). The mean diagnostic accuracy of wearable biosensors based on 10 independent study samples from seven articles (nine for *Se*, eight for *Sp*, six for *Acc*, and eight for *Pre*; Table 10 and Supplementary Table S4) showed an *Se* of 92% (*n* = 205), an *Sp* of 95% (*n* = 195), an *Acc* of 94% (*n* = 169), and a *Pre* of 87% (*n* = 195) [20,23,25,26,28,29,32].

##### Drinking Time

**Figure 5:**
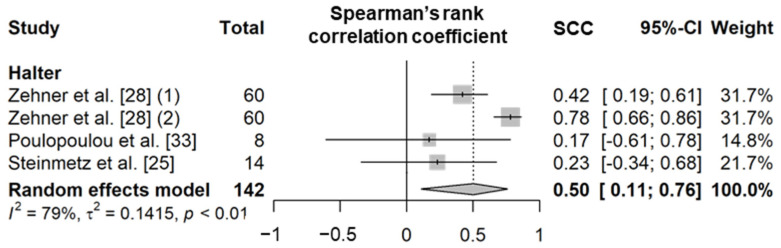
Forest plot of the Spearman’s correlation coefficient of drinking time between wearable sensors and visual observation. Numbers in parentheses indicate individual studies applying different evaluation conditions within the same article. ‘Total’ means the sample size of each study and ‘Weight’ means the weight for the mean based on the sample size.

Drinking time is a variable that represents the amount of time a cow spends drinking water per day. In the literature, drinking behavior is defined as the behavior that cows exhibit when they put their muzzles into water bowls and swallow water [23,24,25,28,33]. The SCC value based on four independent study samples from three articles showed that the drinking time recorded by the sensors was poorly correlated with the actual observations (0.50, *n* = 142; Figure 5 and Supplementary Table S5) [24,25,28,33]. The same sensor product was used for the analysis of drinking time, but there were some differences in the animal type and feeding method (Supplementary Table S5), which showed high heterogeneity (*I*^2^ = 79% and τ^2^ = 0.14). The mean diagnostic accuracy of the wearable biosensors based on four independent study samples from three articles (four for *Se*, *Sp*, *Acc*, and *Pre*; Table 10 and Supplementary Table S6) showed an *Se* of 21.9%, an *Sp* of 99.9%, an *Acc* of 98.8%, and a *Pre* of 30.8% (*n* = 149); notably, *Se* and *Pre* were lower than those relative to other feeding behavior variables [23,25,28].

#### 3.3.2. Activity Behavior

##### Lying Time

**Figure 6:**
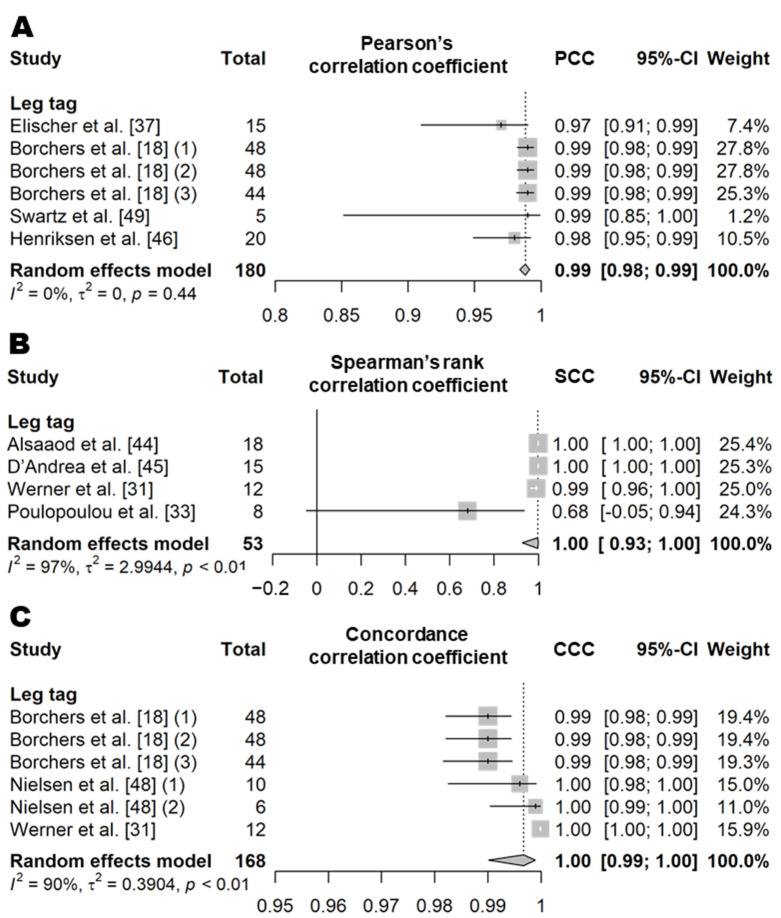
Forest plot of the correlation coefficient of lying time between wearable sensors and visual observation. (**A**–**C**) show Pearson’s correlation coefficient, Spearman’s correlation coefficient, and concordance correlation coefficient, respectively. Numbers in parentheses indicate individual studies applying different evaluation conditions within the same article. ‘Total’ means the sample size of each study and ‘Weight’ means the weight for the mean based on the sample size.

Lying time is a variable that indicates how long an animal is lying on the ground per day. In the literature, lying time is defined as the time during which the body is not supported by the legs and is in contact with the ground [18,31,32,33,37,44,45,46,47,48,49,50]. The PCC and SCC values based on 10 independent study samples from eight articles (six for PCC and four for SCC; Supplementary Table S7) showed that the lying time recorded by the leg tag sensors was very highly correlated with the actual observations (PCC = 0.99, *n* = 180, *I*^2^ = 0%; SCC = 1.00, *n* = 53, *I*^2^ = 97%; Figure 6) [18,31,33,37,44,45,46,49]. The CCC value based on six independent study samples from three articles was also very high (1.00, *n* = 168, *I*^2^ = 90%; Figure 6) [18,31,48]. Both the sensor product and the animal housing condition were different among the studies included in the meta-analysis (Supplementary Table S7), and very high heterogeneity was observed (*I*^2^ = 94% and τ^2^ = 1.69), with the exception of the analysis for PCC. The mean diagnostic accuracy of the wearable biosensors based on five independent study samples from three articles (five for *Se* and *Sp* and four for Pre; Table 10 and Supplementary Table S8) showed an Se of 99.8% (*n* = 53), an *Sp* of 99.9% (*n* = 53), and a *Pre* of 99.9% (*n* = 44) [32,47,50].

##### Standing Time

**Figure 7:**
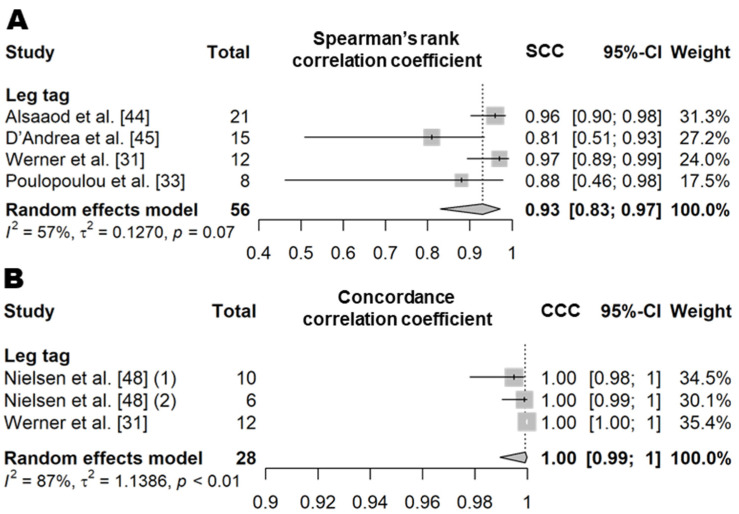
Forest plot of the correlation coefficient of standing time between wearable sensors and visual observation. (**A**,**B**) show Spearman’s correlation coefficient and concordance correlation coefficient, respectively. Numbers in parentheses indicate individual studies applying different evaluation conditions within the same article. ‘Total’ means the sample size of each study and ‘Weight’ means the weight for the mean based on the sample size.

Standing time is a variable that represents the amount of time an animal spends standing per day. In the literature, standing behavior is defined as an animal’s behavior when it is in an upright position with support from the legs but is not walking [31,33,44,45,47,48,50,51]. The SCC value based on four independent study samples from four articles showed that the standing time recorded by the leg tag sensors was very highly correlated with the actual observations (0.93, *n* = 56, *I*^2^ = 57%; Figure 7 and Supplementary Table S9) [31,33,44,45]. In addition, the CCC value based on three independent study samples from two articles was 1.0 (*n* = 28, *I*^2^ = 87%; Figure 7 and Supplementary Table S9) [31,48]. The sensor products and animal housing conditions used were different between the studies included in the meta-analysis of standing time (Supplementary Table S9), and moderate heterogeneity was observed (*I*^2^ = 72% and τ^2^ = 0.63). The mean diagnostic accuracy of wearable biosensors based on four independent study samples from three articles (four for *Se* and *Sp* and three for *Pre*; Table 10 and Supplementary Table S10) showed an *Se* of 95% (*n* = 53), an *Sp* of 98% (*n* = 53), and a *Pre* of 98% (*n* = 44) [47,50,51]. Only one study tested the performance of a neck sensor in estimating the standing time. The reported sensitivity of a neck sensor was approximately 30% lower than that of a leg sensor (*Se* = 63% and *Sp* = 98%) [51].

##### Walking Time

**Figure 8:**
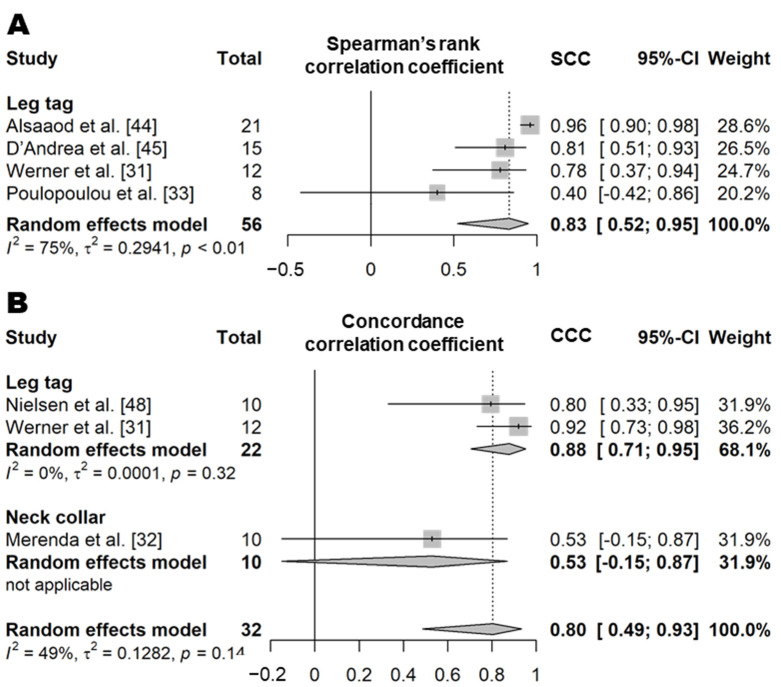
Forest plot of the correlation coefficient of walking time between wearable sensors and visual observation. (**A**,**B**) show Spearman’s correlation coefficient and concordance correlation coefficient, respectively. ‘Total’ means the sample size of each study and ‘Weight’ means the weight for the mean based on the sample size.

Walking time is a variable that represents the amount of time in which the animal walks per day. Walking time is typically defined as a period characterized by at least three consecutive strides in the forward or backward direction [31,32,33,44,45,47,48,50,51]. The SCC value based on four independent study samples from four articles showed that the walking time recorded by the sensors was highly correlated with the actual observations (0.83, *n* = 56, *I*^2^ = 75%; Figure 8 and Supplementary Table S11) [31,33,44,45]. The CCC value based on three independent study samples from three articles was also high (0.80, *n* = 28, *I*^2^ = 49%; Figure 8 and Supplementary Table S11) [31,32,33,44,45,48]. There were differences in the sensor products and the housing conditions used among the studies included in the analysis of the walking time (Supplementary Table S11), but the heterogeneity was moderate (*I*^2^ = 62% and τ^2^ = 0.21). The mean diagnostic accuracy of the wearable biosensors based on five independent study samples from four articles (five for *Se* and *Sp* and four for *Pre*; Table 10 and Supplementary Table S12) showed an *Se* of 34% (*n* = 53), an *Sp* of 98% (*n* = 53), and a *Pre* of 27% (*n* = 44); the *Se* and *Pre* were lower than those relative to other activity behavior variables [32,47,50,51].

##### Step Count

**Figure 9:**
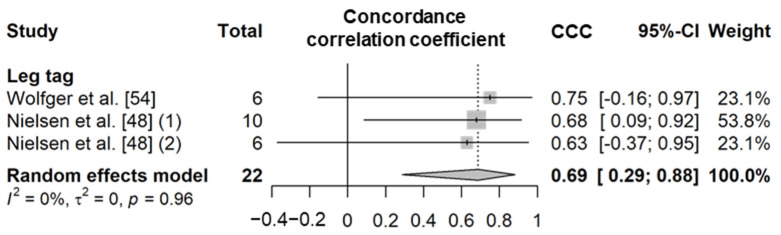
Forest plot of the concordance correlation coefficient of step counts between wearable sensors and visual observation. Numbers in parentheses indicate individual studies applying different evaluation conditions within the same article. ‘Total’ means the sample size of each study and ‘Weight’ means the weight for the mean based on the sample size.

Step count is a variable that represents the number of steps a cow makes per day. A step is defined as the phenomenon occurring when the rear foot is lifted completely off the ground and returned to the ground in any location with or without the movement of the entire body [45,48,52,53,54]. The CCC value based on three independent study samples from two articles showed that the step count measured by the sensors was moderately correlated with the actual observations (0.69, *n* = 22, *I*^2^ = 0%; Figure 9 and Supplementary Table S13) [48,54]. Although there were differences in the sensor product, animal type, and housing condition among the studies included in the analysis of the step counts (Supplementary Table S13), no heterogeneity was observed (*I*^2^ = 0% and τ^2^ = 0).

##### Active Time

**Figure 10:**
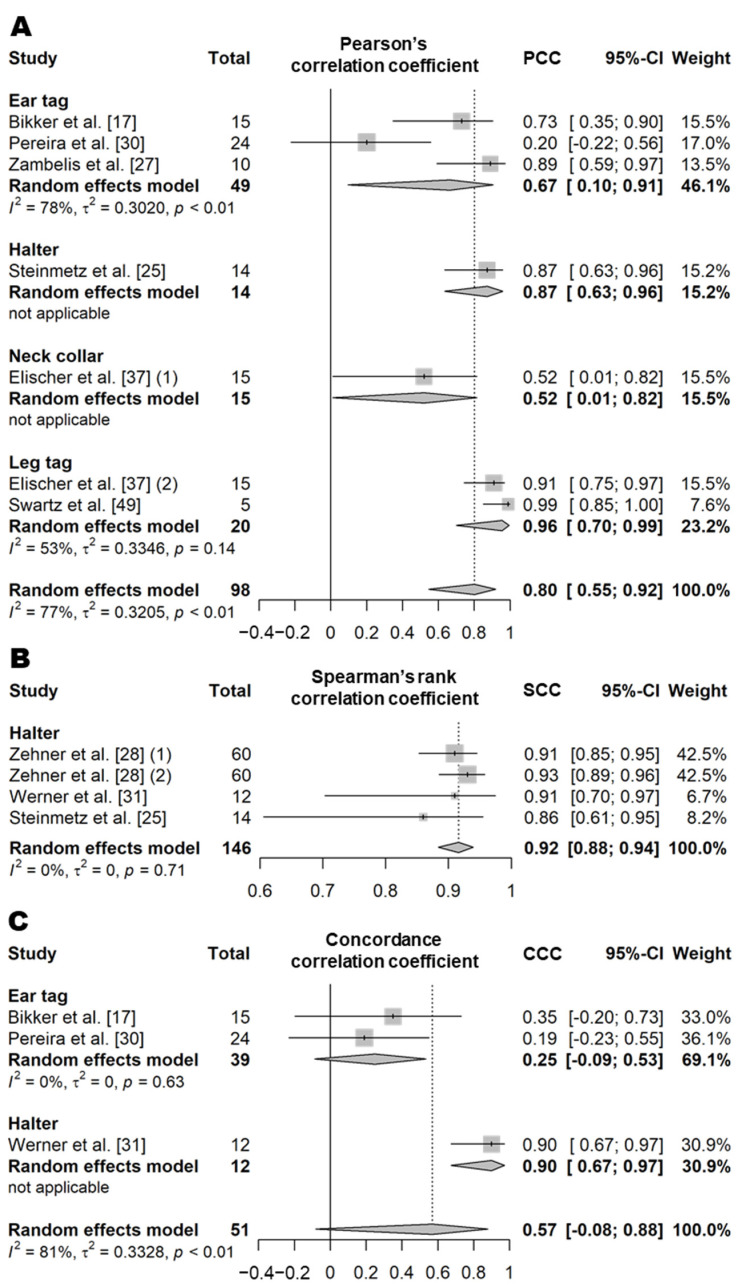
Forest plot of the correlation coefficient of active time between wearable sensors and visual observation. (**A**–**C**) show Pearson’s correlation coefficient, Spearman’s correlation coefficient, and concordance correlation coefficient, respectively. Numbers in parentheses indicate individual studies applying different evaluation conditions within the same article. ‘Total’ means the sample size of each study and ‘Weight’ means the weight for the mean based on the sample size.

Active time is a variable that represents the total active time of a cow per day. It should be noted that the definition of active behavior varies in the literature. Bikker et al. [17] and Pereira et al. [30] defined active behavior as the process of moving the head or body and walking. Elischer et al. [37] defined active behavior as standing or walking behavior. Zambelis et al. [27] defined active behavior in detail as follows: exploring, drinking, urination, defecation, rising, lying down, head swinging, self-grooming, and social interaction. Swartz et al. [49] defined active behavior as a step activity in which the right rear leg is lifted off the floor while standing. The PCC and SCC values based on 10 independent study samples from eight articles (seven for PCC and four for SCC; Supplementary Table S14) showed that the active time recorded by the sensors was highly correlated with the actual observations (PCC = 0.80, *n* = 98, *I*^2^ = 77%; SCC = 0.92, *n* = 146, *I*^2^ = 0%; Figure 10) [17,25,27,28,30,31,37,49]. However, the CCC value based on three independent study samples from three articles showed that such correlation was moderate (0.57, *n* = 51, *I*^2^ = 81%; Figure 10 and Supplementary Table S14) [17,30,31]. There were differences in the sensor products and the housing conditions used between the studies included in the analysis of active time (Supplementary Table S14), and high heterogeneity was observed (*I*^2^ = 79% and τ^2^ = 0.33), with the exception of SCC analysis. Unlike the other sensor types, the halter sensors (RumiWatch Noseband sensors) record active time in terms of movement of the muzzle that is not related to ingestion and drinking [25,28,31]. The active time variables evaluated in these studies showed a high correlation with the actual observed values (PCC = 0.87, SCC = 0.92, and CCC = 0.90) [25,28,31]. The diagnostic accuracy of the halter sensors based on three independent study samples from two articles (three for *Se*, *Sp*, *Acc*, and *Pre*; Table 10 and Supplementary Table S15) showed an *Se* of 93.1%, an *Sp* of 93.4%, an *Acc* of 93.4%, and a *Pre* of 89.9% (*n* = 134) [25,28].

##### Inactive Time (Resting Time)

**Figure 11:**
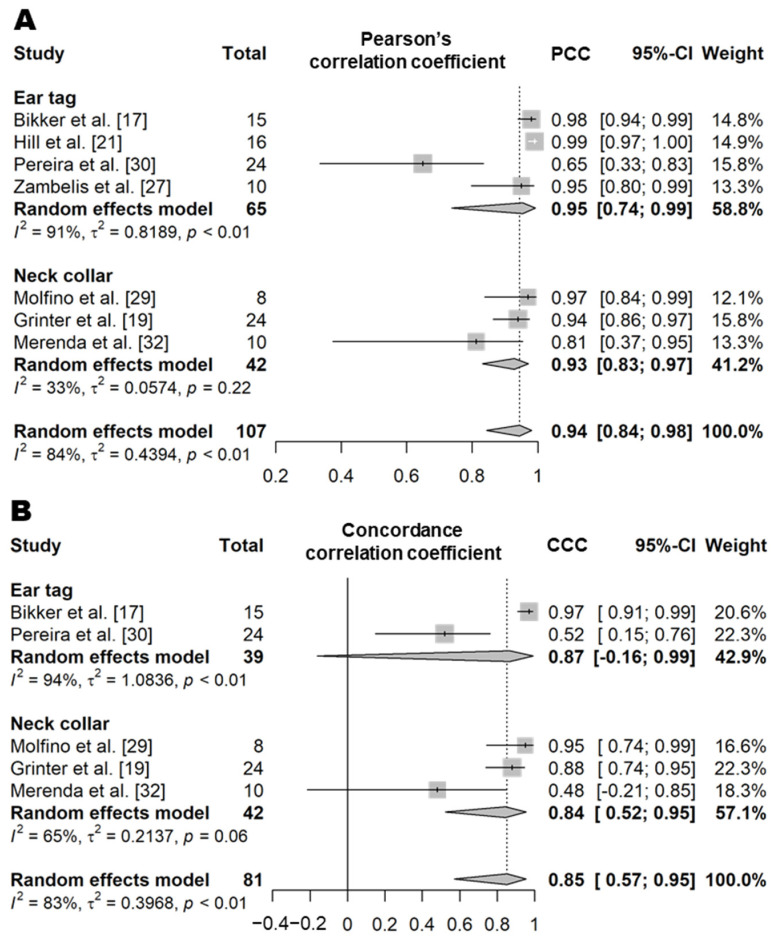
Forest plot of the correlation coefficient of inactive time between wearable sensors and visual observation. (**A**,**B**) show Pearson’s correlation coefficient and concordance correlation coefficient, respectively. Numbers in parentheses indicate individual studies applying different evaluation conditions within the same article. ‘Total’ means the sample size of each study and ‘Weight’ means the weight for the mean based on the sample size.

Inactive or idle time is a variable that represents the amount of time in which cows are not active per day. Inactive time is defined as the time of lying or standing while resting without performing any action, that is, rumination, eating, or drinking [17,19,21,27,29,30,32]. The PCC value based on seven independent study samples from seven articles was very high (0.94, *n* = 107, *I*^2^ = 84%; Figure 11 and Supplementary Table S16) [17,19,21,27,29,30,32]. Although slightly lower than that of the PCC, the CCC value calculated from five independent study samples from five articles was also high (0.85, *n* = 81, *I*^2^ = 83%; Figure 11 and Supplementary Table S16) [17,19,29,30,32]. There were differences in the sensor products used and the animal housing conditions between the studies included in the analysis (Supplementary Table S16), and high heterogeneity was observed (*I*^2^ = 84% and τ^2^ = 0.42). The mean diagnostic accuracy of the wearable biosensors based on three independent study samples from two articles (three for *Se*, *Sp*, and Pre; Table 10 and Supplementary Table S17) showed an Se of 59% (*n* = 53), an *Sp* of 98% (*n* = 53), and a *Pre* of 89% (*n* = 44) [29,32].

#### 3.3.3. Rumen Status

**Figure 12:**
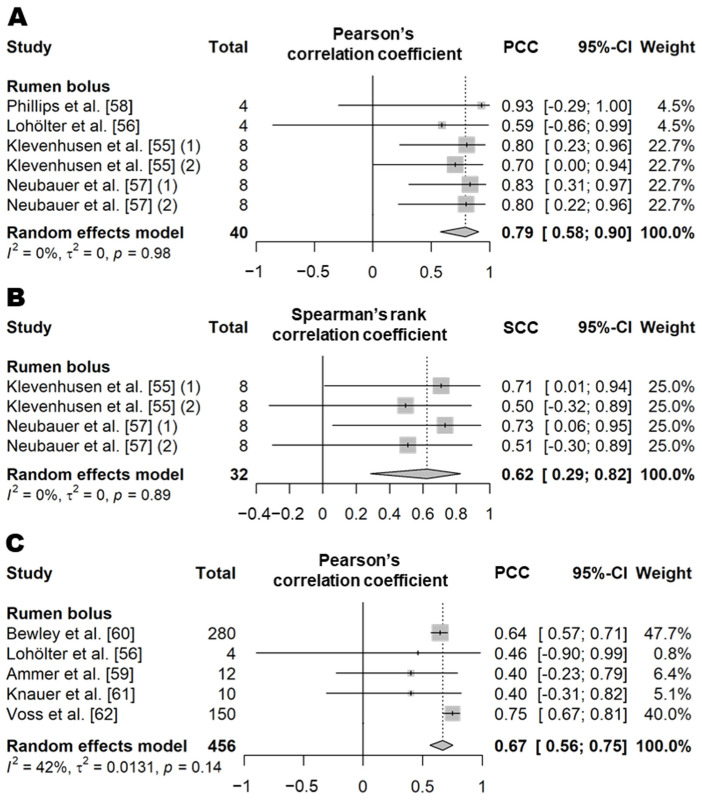
Forest plot of the correlation coefficient of rumen status (pH and temperature) between wearable sensors and visual observation. (**A**,**B**) show Pearson’s correlation coefficient and concordance correlation coefficient of rumen pH, respectively. (**C**) shows the Pearson’s correlation coefficient of rumen temperature. Numbers in parentheses indicate individual studies applying different evaluation conditions within the same article. ‘Total’ means the sample size of each study and ‘Weight’ means the weight for the mean based on the sample size.

Rumen pH and rumen temperature are variables measured using reticulo-rumen bolus sensors. In the case of rumen pH measured by the bolus sensors, the pH of the rumen fluid measured by a pH meter is used as the gold standard [55,56,57,58]. The PCC value of the correlation between the pH measured by these sensors and actual observations, based on six studies from four articles, was high (0.79, *n* = 40, *I*^2^ = 0%; Figure 12) [55,56,57,58]. However, the CCC value based on two articles (four independent studies) indicated an only moderate correlation (0.62, *n* = 32, *I*^2^ = 0%; Figure 12) [55,57]. There were differences in the sensor product and gold standard used between the studies included in the analysis (Supplementary Table S18), but heterogeneity was not observed (*I*^2^ = 0% and τ^2^ = 0). In the literature, the rumen temperature measured by the bolus sensors was compared with the rectal temperature measured using digital thermometers [56,59,60,61,62]. The PCC value from five articles (contributing to five independent study samples) showed that the rumen temperature measured by the bolus sensors was moderately correlated with the actual observations (PCC = 0.67, *n* = 456; Figure 12) [56,59,60,61,62]. There were differences in the sensor products between studies included in the analysis (Supplementary Table S18), but low heterogeneity was observed (*I*^2^ = 42% and τ^2^ = 0.01).

**Table 10:** Meta-analysis results of diagnostic accuracy of feeding and activity behavior variables from wearable sensors.

	Diagnostic Accuracy ^1,2^											
	Sensitivity			Specificity			Accuracy			Precision		
Variable	Study No.	*n*	% (95% CI)	Study No.	*n*	% (95% CI)	Study No.	*n*	%(95% CI)	Study No.	*n*	% (95% CI)
Feeding behavior												
Eating time	10	220	84.9 (70.0–92.7)	9	210	96.3 (91.7–98.4)	7	184	90.8 (86.3–93.9)	9	210	87.3 (72.9–94.3)
Ruminating time	9	205	92.2 (85.6–95.9)	8	195	95.4 (91.0–97.7)	6	169	93.9 (91.0–95.1)	8	195	87.0 (77.7–92.5)
Drinking time	4	149	21.9 (5.5–37.1)	4	149	99.9 (99.7–100)	4	149	98.8 (98.0–99.3)	4	149	30.8 (15.0–45.1)
Activity behavior												
Lying time	5	53	99.8 (98.2–100)	5	53	99.9 (99.6–100)	-	-	-	4	44	99.9 (96.6–100)
Standing time	4	38	95.3 (87.9–98.2)	4	38	98.3 (94.7–99.4)	-	-	-	3	29	97.9 (86.7–99.7)
Walking time	5	48	33.8 (1.1–60.0)	5	48	98.0 (96.0–99.0)	-	-	-	4	39	26.6 (10.5–57.1)
Active time	3	134	93.1 (90.3–95.1)	3	134	93.4 (90.8–95.3)	3	134	93.4 (90.7–95.3)	3	134	89.9 (85.7–92.9)
Inactive time	3	28	59.2 (22.7–81.1)	3	28	98.2 (95.6–99.3)	-	-	-	3	28	89.3 (75.7–95.5)

^1^ Study No.: number of studies; evaluation results analyzed under different conditions within the same article are counted as individual studies. ^2^ *n*, sample size; number of animals.

## 4. Summary and Implications

A wide variety of wearable wireless biosensor systems for health or estrus detection are currently available in the market. Most of these sensor systems measure acceleration using a three-axis accelerometer and convert this into a numeric value to quantify specific physiological parameters, such as eating time, rumination time, and resting time, using a customized algorithm. The reporting methods (reporting frequency, data units, etc.) of the information generated by the sensors are also diverse. Important basic information on the sensors, such as the frequency of data measurement and the algorithm used for calculating the value of a specific variable from acceleration, was largely undisclosed because of company confidentiality.

To date, several studies have evaluated different parameters related to feeding behavior, moving behavior, and rumen status that were measured and calculated using sensor systems. These sensor systems showed a high performance in measuring most of the physiological parameters. However, the sensor performance for some parameters (e.g., drinking time and walking time) needs to be improved [23,24,25,28,32,47,50,51], and a specific sensor showed low performance for a particular behavior (i.e., walking time measured with a neck sensor) [32,51]. Moreover, it seems that the mounting position of a sensor using an accelerometer is critical to detect a cow’s specific behavior of interest, which is consistent with a previous report [63]. In particular, feeding behavior was classified more accurately by a neck-mounted than a leg-mounted accelerometer (*Se* 96 versus 80% and *Pre* 88 versus 79%, respectively), but the opposite was true for lying behavior (*Se* 95 versus 96% and *Pre* 82 versus 97%, respectively) [63].

A standardized guideline for reporting sensor evaluation is required. Different performance levels were reported under different conditions, which was reflected in the considerable heterogeneity of the meta-analysis (average *I*^2^ = 76%). In some cases, the same brand of sensor was evaluated very differently in the literature, even under the same feeding and housing conditions [18,22,27,32,36]. Unfortunately, a number of literature sources provided insufficient evaluation criteria, which makes it impossible to ascertain which evaluation factor caused such differences in performance between the sensors. In order to clarify the factors affecting the difference in the accuracy of these sensors, more detailed information is required as follows: animal information (species, gender, physiological status, etc.), housing information (stall type, pen size, stocking density, etc.), data information (observation time per animal, number of observation points per day, total collection days, etc.), and gold-standard information (method, reliability within and between observers, etc.). In the medical field, there is a guideline for writing papers that report the accuracy of a diagnostic method called a Standards for Reporting of Diagnostic Accuracy (STARD) statement [64]. This guideline contains a list of essential reporting items that can be used as a checklist to ensure that a report of a diagnostic accuracy study contains the necessary information. Performing a meta-analysis using articles written using this guideline enables a detailed discussion of bias and heterogeneity among the studies. Therefore, it is necessary to establish reporting guidelines including the above-mentioned factors (i.e., animal, housing, gold standard, etc.), such as the STARD statement, for papers reporting the accuracy of wearable wireless biosensors.

## 5. Conclusions

In conclusion, the present study showed that the wearable biosensors tested in the literature predict targeted behavioral information with high accuracy. However, the algorithms used to generate some types of information, such as drinking time and walking time, need to be improved. Furthermore, since the accuracy of behavioral information changes sensitively depending on the evaluation conditions, it is recommended to evaluate each sensor using adequate and validated criteria and report the evaluation criteria in detail.

## Figures and Tables

**Table 9:** Information about currently available tail- and vaginal-mounted type sensor.

Product	Company (Parent Company)	Country	Management Software	Mobile Application	Dimensions (mm × mm)	Weight (g)	Battery Life	Range (m)	Built-In Sensors	Detection
Tail										
Smart’Vel	Evolution international	FR	×	×	-	75	5 years	-	Accelerometer	Calving
Alert’Vel	ALB Innovation	FR	×	×	-	-	-	2000	Accelerometer	Calving
Moocall Calving Sensor	Moocall Ltd.	IE	Moocall Breedmanager	○	-	-	60 days	-	Accelerometer	Calving
Vagina										
Vel’Phone	Medria Inc.	FR	Farm’Life^®^ (Vel’Live^®^)	○	116 × 26	87	-	1000	Temperature sensor	Health disorder/Calving
Cow Call	Cow Call	IE	×	×	-	-	2 years	-	Temperature sensor Light sensor	Calving

## Data Availability

Data is contained within the article and Supplementary Materials.

## Supplementary Materials

The following are available online at https://www.mdpi.com/article/10.3390/ani11102779/s1, Table S1: Evaluation results (correlation coefficient) for eating time (time spent eating) among feeding behavior variables generated by the sensors, Table S2: Evaluation results (diagnostic accuracy) for eating time (time spent eating) among feeding behavior variables generated by the sensors, Table S3: Evaluation results (correlation coefficient) for rumination time (time spent ruminating) among feeding behavior variables generated by the sensors, Table S4: Evaluation results (performance) for rumination time (time spent ruminating) among feeding behavior variables generated by the sensors, Table S5: Evaluation results (correlation) for drinking time (time spent for drinking) among feeding behavior variables generated by the sensors, Table S6: Evaluation results (performance) for drinking time (time spent for drinking) among feeding behavior variables generated by the sensors, Table S7: Evaluation results (correlation) for lying time (time spent lying) among activity behavior variables generated by the sensors, Table S8: Evaluation results (performance) for lying time (time spent lying) among activity behavior variables generated by the sensors, Table S9: Evaluation results (correlation) for standing time (time spent standing) among activity behavior variables generated by the sensors, Table S10: Evaluation results (performance) for standing time (time spent standing) among activity behavior variables generated by the sensors, Table S11: Evaluation results (correlation) for walking time (time spent walking) among activity behavior variables generated by the sensors, Table S12: Evaluation results (performance) for walking time (time spent walking) among activity behavior variables generated by the sensors, Table S13: Evaluation results (correlation) for step counts (the number of steps) among activity behavior variables generated by the sensors, Table S14: Evaluation results (correlation) for active time (time spent activity) among activity behavior variables generated by the sensors, Table S15: Evaluation results (performance) for active time (time spent activity) among activity behavior variables generated by the sensors, Table S16: Evaluation results (correlation) for inactive time (time spent inactivity) among activity behavior variables generated by the sensors, Table S17: Evaluation results (performance) for inactive time (time spent inactivity) among activity behavior variables generated by the sensors, Table S18: Evaluation results (correlation) for rumen pH and rumen temperature generated by the reticulo-rumen bolus sensors.
